# The path to growth resilience: A serial mediation model linking temporal coherence to goal clarity and cognitive curiosity

**DOI:** 10.1371/journal.pone.0343699

**Published:** 2026-03-25

**Authors:** Juan He, Hao Jin

**Affiliations:** 1 School of Foreign Studies, Guangxi University of Science and Technology, LiuZhou, GuangXi, China; 2 Jiangxi Tellhow Animation College, Nanchang, Jiangxi, China; De La Salle University - Dasmariñas Campus: De La Salle University - Dasmarinas Campus, PHILIPPINES

## Abstract

Fostering growth resilience is critical for college students to succeed and develop amidst academic and social environment. Temporal coherence — a balanced integration of past, present, and future perspectives — is recognized as important, yet the mechanisms linking it to growth resilience remain unclear. This study aimed to examine the effect of temporal coherence on growth resilience among college students and to test the serial mediating roles of goal clarity and cognitive curiosity. The study adopted a cross-sectional research design and administered an anonymous questionnaire to 452 Chinese college students (50.4% male and 49.6% female) via an online survey platform. The measures included the Zimbardo Time Perspective Inventory (ZTPI), the Goal Clarity Scale (GCS), the Cognitive Curiosity Scale, and the Chinese version of the Connor-Davidson Resilience Scale (CD-RISC). Hypothesis testing was conducted using Structural Equation Modelling (SEM) and the bias-corrected bootstrap method. The results indicated significant positive correlations among temporal coherence, goal clarity, cognitive curiosity, and growth resilience (r = 0.56–0.71, p < 0.01).. Structural equation model demonstrated good fit (χ²/df = 2.41, CFI = 0.961, TLI = 0.954, RMSEA = 0.056). Temporal coherence directly influenced growth resilience (β = 0.22, p = 0.002) and also exerted indirect effects through three pathways: via goal clarity alone (effect = 0.12), via cognitive curiosity alone (effect = 0.10), and via the serial mediation of goal clarity then cognitive curiosity (effect = 0.15). The serial mediated path accounted for 25.4% of the total effect, which was the indirect path with the largest effect size. Therefore, temporal coherence is an important predictor of growth resilience, primarily mediated through the sequential mechanism of enhancing goal clarity, which in turn stimulates cognitive curiosity.. This study reveals an intrinsic pathway from coherent time perception to resilient growth and highlights that interventions targeting time perspective, goal setting, and curiosity stimulation can effectively promote resilience in educational practice.

## 1 Introduction

Amid rapid social change, economic fluctuations, and sustained academic pressures, the psychological adaptation of college students has garnered significant attention from researchers and practitioners alike [1]. The transition to adulthood, combined with a competitive educational and future job market, subjects students to considerable stress, potentially undermining their psychological well-being and academic persistence [2]. Within this context, growth resilience—defined as the capacity to not only recover from adversity but also to learn, adapt, and emerge stronger—is a key determinant of student success and long-term development [3]. Consequently, identifying factors that foster this capacity has become a critical research objective..

Individuals’ adaptive functioning is deeply rooted in their temporal orientation—their perception of and connection to the past, present, and future. Building on the seminal work of Zimbardo and Boyd [3] research on time perspective has demonstrated its profound impact on decision-making, well-being, and life outcomes [4]. A temporally coherent profile—characterized by the balanced and flexible integration of a positive past, a hedonically mindful present, and a future-oriented perspective—is considered the most adaptive [Guo, et al. 2025]. Individuals with a high degree of temporal coherence are assumed to possess a stable psychological platform with which to cope with life’s challenges. However, while empirical studies consistently link this balanced temporal profile to positive outcomes (e.g., life satisfaction), the specific mechanisms through which it fosters the kind of robust, growth-oriented resilience required in contemporary settings remain under-explored [5].

One key mechanism connecting temporal coherence and resilience may be goal clarity. Clear, well-defined goals provide direction and meaning, translating a broad temporal perspective into a concrete roadmap for action [5]. A balanced view of time likely facilitates this clarity, which allows individuals to constructively draw on the past, enjoy the present without being bound by it, and purposefully work toward future goals. Prior research has established goal clarity as a predictor of academic achievement and motivation [6]. However, it has received less attention as a potential mediating variable that translates broad temporal perceptions into specific motivational states that precede resilience, as goal clarity alone may not fully explain how individuals sustain effort in the face of enduring difficulties.

We propose that the link from goal clarity to growth resilience is critically energized by cognitive curiosity – an intrinsic drive for knowledge and cognitive exploration [7]. When individuals have clear goals, the path to achieving those goals often presents puzzles and challenges. Strong cognitive curiosity motivates individuals to tackle these challenges for the intrinsic rewards that come with understanding itself, thus promoting deeper information processing and more adaptive problem solving [8]. This positive, curiosity-driven engagement with adversity is a hallmark of the growth resilience process. Although curiosity is widely recognised as a facilitator of learning, its specific function as a sequential mechanism activated by a clear goal and thus driving resilience within the framework of temporal psychology has not been fully tested.

Therefore, this study aims to integrate these lines of research by proposing and testing a serial mediation model.Based on the above theoretical derivations, the following research hypotheses are proposed:

Hypothesis 1 (H1): Temporal coherence positively predicts goal clarity.

Hypothesis 2 (H2): Temporal coherence positively predicts cognitive curiosity.

Hypothesis 3 (H3): Temporal coherence positively predicts growth resilience.

Hypothesis 4 (H4): Goal clarity and cognitive curiosity sequentially mediate the relationship between temporal coherence and growth resilience. Specifically, temporal coherence enhances goal clarity, which in turn fosters cognitive curiosity, ultimately leading to greater growth resilience.

**Fig 1:**
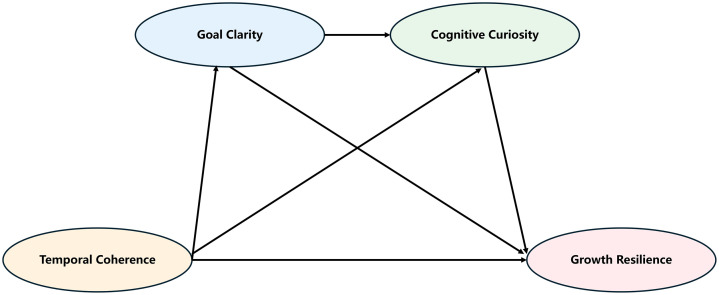
Theoretical model diagram.

This model extends current understanding by integrating time perspective theory, goal-setting theory, and cognitive motivation theory into a cohesive framework. It elucidates the sequential psychological pathway through which temporal coherence may contribute to resilience, moving beyond isolated variable examinations to a more holistic view of adaptive functioning. The present study thus not only contributes to the theoretical integration of the psychology of time, the science of motivation, and positive psychology, but also provides viable insights into educational interventions aimed at developing resilient individuals who are able to flourish in a complex world, as shown in Fig 1.

## 2 Methodology

### 2.1 Participants and procedures

This study employed a cross-sectional design. Participants were recruited from five public universities in China using a convenience sampling method, with efforts made to ensure diversity across major disciplines (i.e., humanities, social sciences, sciences, and engineering). All participants were informed of the purpose, content, and rights of the study through an electronic informed consent form and ticked the “Agree to Participate” box on the first page of the questionnaire before entering the formal questionnaire. Data were collected anonymously and without any personal identifiable information. The questionnaire was distributed through the “Questionnaire Star” platform, with IP address restriction (the same IP can only fill in the questionnaire once) and time filtering (less than 300 seconds is regarded as invalid) to ensure the quality of the data.

A total of 500 undergraduates were invited to participate. After excluding questionnaires with incomplete data, regular responses and completion time less than 300 seconds, 452 valid questionnaires were retained, with an effective recovery rate of 90.4%. The sample consisted of 228 male (50.4%) and 224 female (49.6%) students. The grade distribution was 27.4% for freshmen, 25.7% for sophomores, 28.1% for juniors, and 18.8% for seniors. In terms of academic background, 28.3% were from the humanities and social sciences, and 56.7% were from science and engineering.The remaining 15.0% may represent undeclared or other disciplines Data were collected between May and September 2025. We note that the convenience sampling method may limit the generalizability of the findings, and future research could employ random sampling to enhance representativeness.

### 2.2 Measurement instruments

All instruments used in this study were mature scales, with Chinese versions translated and adapted where necessary. All scales were scored on a 5-point Likert scale (1 = strongly disagree, 5 = strongly agree).

#### 2.2.1 Temporal coherence.

Temporal coherence was measured using a 15-item scale adapted from the Zimbardo Time Perspective framework [9], which measures temporal coherence by assessing the degree to which an individual is coordinated across three temporal dimensions: past, present and future. The scale is scored on a 5-point Likert scale ranging from “not at all” to “completely”, with higher total scores indicating better temporal coordination. The Cronbach’s alpha coefficient of the scale was 0.87 in this study.

#### 2.2.2 Goal clarity.

The Goal Clarity Scale (GCS) [10] with 14 entries was used. Sample entries include “I know exactly what I want to achieve through my university education” and “I have a clear plan for my career development in the next five years”. The Cronbach’s alpha coefficient for the scale in this study was 0.91.

#### 2.2.3 Cognitive curiosity.

The Cognitive Curiosity Scale [11] with 16 entries was used. Sample entries included, “When encountering new concepts, I like to probe deeper into their meanings,” and “Complex questions motivate me to find answers.” The Cronbach’s alpha coefficient for the scale in this study was 0.89.

#### 2.2.4 Growth resilience.

Growth resilience was measured using the 25-item Chinese version of the Connor-Davidson Resilience Scale (CD-RISC) [12] with 25 entries was used. Sample entries included “I tend to become stronger after experiencing adversity” and “I can adapt to changes in my life”. The Cronbach’s alpha coefficient of the scale in this study was 0.93.

### 2.3 Data analysis

Data analysis followed the two-step method recommended by Anderson and Gerbing [12]. First,confirmatory factor analysis (CFA) was conducted using AMOS 26.0 to test the validity and discriminant validity of the measurement model. Model fit was assessed by several indices: χ²/df, Comparative Fit Index (CFI), Tucker-Lewis Index (TLI), Root Mean Square Error of Approximation (RMSEA), and Standardized Root Mean Square Residual (SRMR).

Second, structural equation modeling (SEM) with maximum likelihood estimation was used to test the hypothesised serial mediation model. Indirect effects were tested using the bias-corrected bootstrap method with 5,000 bootstrap samples and 95% confidence intervals. This method is considered robust in testing for mediation effects as it does not assume normality of the sampling distribution [12].

To assess common method bias, a Harman one-way test was conducted. The results showed that the first factor explained 32.7% of the variance, which is below the critical value of 40%, indicating that common method bias was not a serious problem in this study [13].

### 2.4 Ethical compliance statement

This study involved an anonymous online survey that collected no sensitive or personally identifiable information and posed minimal risk to participants. In accordance with Article 32 of the *Measures for Ethical Review of Life Science and Medical Research Involving Humans* (National Health Commission, 2023), it qualified for exemption from formal ethical board review because it (i) employed observational methods without interference, and (ii) used strictly anonymized data.

Nevertheless, ethical principles were rigorously upheld. All participants were presented with a detailed information sheet on the first page of the online questionnaire, outlining the study’s purpose, data usage, and privacy protections. Electronic informed consent was obtained by requiring participants to check an “Agree to Participate” box before proceeding.The ethical guidelines of the Declaration of Helsinki were followed to ensure that the rights of the participants were fully protected.

## 3 Results

### 3.1 Descriptive statistics and common method bias test

The study began with a descriptive statistics analysis of the 452 valid responses. As shown in Table 1, the mean scores for all variables ranged from 3.65 to 4.02 on the 5-point scale, indicating moderately high levels across constructs. Cognitive curiosity had the highest mean score (M = 4.02, SD = 0.63), followed by growth resilience (M = 3.91, SD = 0.59) and temporal coherence (M = 3.82, SD = 0.71). Goal clarity had the lowest mean score (M = 3.65, SD = 0.68).

**Table 1:** Descriptive statistics and correlation analysis matrix (N = 452).

Variables	M	SD	Skewness	Kurtosis	1	2	3	4
**1. Temporal Coherence**	3.821	0.712	−0.324	0.152	1			
**2. Goal Clarity**	3.652	0.681	−0.251	0.083	0.564**	1		
**3. Cognitive Curiosity**	4.023	0.634	−0.412	0.224	0.631**	0.593**	1	
**4. Growth Resilience**	3.912	0.591	−0.378	0.168	0.708**	0.672**	0.753**	1

* p < 0.05, ** p < 0.01.

Pearson correlation analyses (Table 1) revealed significant positive bivariate correlations among all variables (p < .01). The strongest correlation was between temporal coherence and growth resilience (r = .71, p < .01). All correlation coefficients were below.75, suggesting no severe multicollinearity concerns.

The results of exploratory factor analysis showed that the variance explained by the unrotated first factor was 32.7%, which was lower than the critical criterion of 40%. Furthermore, a confirmatory factor analysis comparing a single-factor model (all items loading on one factor) with the hypothesized four-factor model showed markedly worse fit for the single-factor model (χ²/df = 5.89, CFI = .72, TLI = .68, RMSEA = .121), providing additional evidence against substantial common method bias.

### 3.2 Measurement model validation

**Fig 2:**
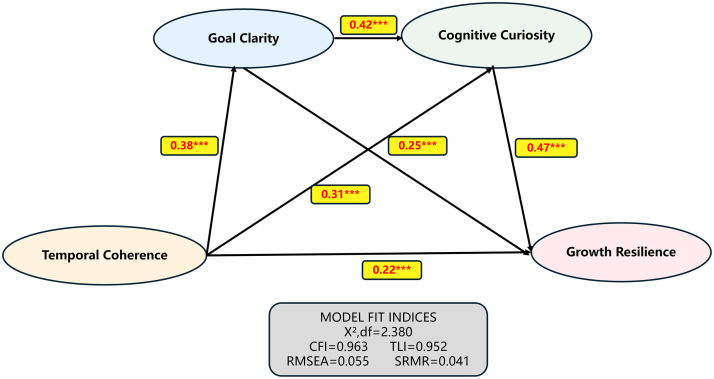
Chain-mediated model.

We conducted a confirmatory factor analysis (CFA) to evaluate the measurement model. As shown in Table 2, all fit indices of the four-factor model met the desirable criteria: χ²/df = 2.38 (<3), CFI = 0.963 (>0.90), TLI = 0.952 (>0.90), RMSEA = 0.055 (<0.08), and SRMR = 0.041 (<0.08). Compared to other competing models, the four-factor model had significantly better goodness of fit, supporting the discriminant validity of the theoretical constructs in this study, as shown in Fig 2.

**Table 2:** Comparison of validated factor analysis models (N = 452).

Model	χ²	df	χ²/df	CFI	TLI	RMSEA	SRMR
**Four-factor model**	688.451	289	2.382	0.963	0.952	0.055	0.041
**Three-factor model**	1125.892	295	3.816	0.872	0.851	0.098	0.087
**Two-factor model**	1569.124	299	5.247	0.761	0.732	0.124	0.113
**Single factor model**	2148.335	302	7.113	0.645	0.608	0.151	0.136

The analysis of composite reliability (CR) and average variance extracted (AVE) further validated the reliability of the measurement model. As shown in Table 3, the CR values of all variables ranged from 0.83 to 0.91, exceeding the criterion of 0.70, and the AVE values ranged from 0.58 to 0.63, exceeding the criterion of 0.50, which indicated that the scales had good combined reliability and convergent validity.

**Table 3:** Results of reliability and validity analysis.

Variable	Number of questions	CR	AVE	Cronbach’s α
**1. Temporal Coherence**	15	0.853	0.608	0.872
**2. Goal Clarity**	14	0.881	0.582	0.913
**3. Cognitive Curiosity**	16	0.834	0.591	0.889
**4. Growth Resilience**	25	0.912	0.627	0.934

**CR = Composite Reliability; AVE = Average Variance Extracted.**

The results of the test of discriminant validity show (see Table 4) that the square root of AVE for each variable is greater than the correlation coefficients between that variable and the other variables, which satisfies the Fornell-Larcker criterion and further confirms the discriminant validity between the constructs.

**Table 4:** Distinguishing validity test: square root of AVE and correlation coefficient matrix.

Variables	1	2	3	4
**1. Temporal Coherence**	**0.780**			
**2. Goal Clarity**	0.564	**0.763**		
**3. Cognitive Curiosity**	0.631	0.593	**0.769**	
**4. Growth Resilience**	0.708	0.672	0.753	**0.792**

**Bolded diagonal values are square roots of AVE.**

### 3.3 Structural modelling and hypothesis testing

On the basis of validating the measurement model, we used structural equation modelling to test the research hypotheses. The fit metrics of the structural model showed good model fit: χ²/df = 2.41, CFI = 0.961, TLI = 0.954, RMSEA = 0.056, and SRMR = 0.043.

The results of the path analyses (see Table 5) showed that all hypothesised paths reached the level of significance. Specifically, temporal coherence was significantly positively associated with goal clarity (β = 0.38, p < 0.001), supporting H1; the direct effect of temporal coherence on cognitive curiosity was also significant (β = 0.25, p < 0.001), supporting H2; goal clarity was significantly positively associated with cognitive curiosity (β = 0.42, p < 0.001); both goal clarity (β = 0.31, p < 0.001) and cognitive curiosity (β = 0.47, p < 0.001) was significantly positively associated with growth resilience; and after controlling for mediator variables, the direct effect of temporal coherence on growth resilience was still significant (β = 0.22, p = 0.002), suggesting the presence of partial mediation effects.

**Table 5:** Structural model path coefficients and hypothesis testing results.

Research hypotheses and path relationship	Standardised coefficients	Unstandardised coefficients	SE	CR	p	Conclusion
**H1: Temporal coherence→ Goal Clarity**	0.382	0.361	0.042	6.124	<0.001	Support.
**H2: Temporal Coherence → Cognitive Curiosity**	0.253	0.228	0.051	4.332	<0.001	Support.
**Goal Clarity→ Cognitive Curiosity**	0.418	0.392	0.063	5.873	<0.001	–
**Goal Clarity → Growth Resilience**	0.312	0.274	0.071	3.981	<0.001	–
**Cognitive Curiosity → Growth Resilience**	0.473	0.441	0.052	7.214	<0.001	–
**H3:Temporal coherence → Growth Resilience (direct effect)**	0.224	0.183	0.062	3.121	0.002	–

### 3.4 Analysis of chained mediating effects

To test the serial mediating effects of goal clarity and cognitive curiosity, we examined indirect effects using the bias-corrected bootstrap method with 5,000 samples. As shown in Table 6, the total effect was 0.59, with a direct effect of 0.22 (37.3% of the total effect) and a total indirect effect of 0.37 (62.7% of the total effect), indicating that the mediating effect plays an important role in explaining the relationship of the variables.

**Table 6:** Decomposition table of chained mediation effects.

Type of effect and path	Effect Value	Boot SE	95% confidence interval	Effect Percentage	Significance
**Total effect**	0.592	0.072	[0.461, 0.724]	100.0%	Significant
**Direct effect**	0.224	0.062	[0.102, 0.346]	37.8%	Significant
**Total indirect effect**	0.368	0.051	[0.282, 0.473]	62.2%	Significant
**Indirect Path 1: Temporal coherence → Goal Clarity → Growth Resilience**	0.119	0.032	[0.072, 0.192]	20.1%	Significant
**Indirect Path 2: Temporal coherence→ Cognitive curiosity → Growth Resilience**	0.102	0.024	[0.062, 0.153]	17.2%	Significant
**Indirect Path 3: Temporal coherence → Goal Clarity → Cognitive curiosity → Growth Resilience**	0.147	0.038	[0.081, 0.231]	24.8%	Significant.

Specifically for the three indirect effect pathways: the first pathway “temporal coherence → goal clarity → growth resilience” had an effect size of 0.12 (95% CI [0.07, 0.19]); the second pathway “temporal coherence → cognitive curiosity → growth resilience” had an effect size of 0.10 (95% CI [0.06, 0.15]); and the third chained mediation pathway “temporal coherence → goal clarity → cognitive curiosity → growth resilience” had an effect size of 0.15 (95% CI [0.06, 0.15]). The third chain-mediated pathway, ‘Temporal coherence → Goal clarity → Cognitive curiosity → Resilience’, had an effect size of 0.15 (95% CI [0.08, 0.23]), and the confidence intervals for all three pathways did not include zero, indicating a significant mediation effect. The chain mediation path had the largest effect size, accounting for 25.4% of the total effect, supporting hypothesis H4.

## 4 Discussion

This study systematically examined the mechanism of the influence of temporal coherence on college students’ growth resilience by constructing a chain mediation model. The results support all the research hypotheses and reveal that time coherence not only is directly associated with growth resilience, but more importantly, it is a psychological mechanism that works through the sequential mediation pathway of “goal clarity-cognitive curiosity”. The theoretical and empirical findings are discussed in depth below.

### 4.1 The direct effect of temporal coherence on growth resilience and its mediating pathway

The findings confirmed that temporal coherence is a significant positive predictor of growth resilience (β = .22, p = .002), supporting Hypothesis 3. This finding is consistent with Zimbardo and Boyd’s [14] theory of temporal perspective, suggesting that a balanced temporal perspective can indeed provide individuals with cognitive resources to cope with adversity. Individuals with a high degree of temporal coherence are able to flexibly switch between past experiences, present enjoyment, and future planning, and this temporal balance allows them to remain psychologically resilient in the face of setbacks, avoiding falling into the cognitive rigidity that may result from a single temporal perspective [13].

More importantly, this study found three significant mediating pathways. The substantial total indirect effect (accounting for 62.7% of the total effect) suggests that the influence of temporal coherence on growth resilience operates predominantly through mediating mechanisms, rather than directly. Among them, the chain mediation path “time coherence→goal clarity→cognitive curiosity→growth resilience” has the largest effect value (0.15, accounting for 25.4%), which fully verifies the hypothesis H4, and this finding has important theoretical significance, which reveals that the transformation process from time cognition to mental resilience is an orderly chain of mental processing: a balanced view of time helps individuals establish clear goals, which in turn stimulates cognitive exploration and ultimately enhances the ability to grow in adversity.[14].

### 4.2 The critical mediating role of goal clarity

Goal clarity plays a key mediating role between temporal coherence and growth resilience, not only in the direct path (effect size 0.12), but also in the chain path. This finding supports the goal-setting theory of Locke and Latham [15] and provides an important extension of it. Specifically, goal clarity appears to translate a balanced temporal perspective into a concrete system of objectives, thereby providing direction and meaning that sustains perseverance during adversity. When individuals have coherent perceptions of the past, present, and future, they are more likely to develop an internally consistent goal system, which serves as a “psychological anchor” to prevent individuals from losing their way in the face of adversity [16].

Notably, the significant predictive effect of goal clarity on cognitive curiosity (β = 0.42) suggests that clear goals not only provide direction, but also stimulate individuals’ desire to explore the path to achieve the goals. This finding departs from the traditional “ends-means” view of rationality and suggests that goal setting may influence individual behaviour by stimulating intrinsic cognitive motivation, which provides a new perspective on understanding the relationship between goals and behaviour [17].

### 4.3 The core driving role of cognitive curiosity

This study found that cognitive curiosity was the most significant predictor of growth resilience (β = 0.47), a result that has important theoretical value. It suggests that in the face of adversity, cognitive balance and goal clarity alone may not be sufficient to lead to true growth, and that the desire for active exploration and curiosity represented by cognitive curiosity is the core driver that propels individuals to achieve transcendent growth. This finding echoes that of Kashdan and Silvia [18], confirming the critical position of cognitive curiosity in the process of individual adaptation and development.

The place of cognitive curiosity in the model is also quite revealing. It is both an important mediator of goal clarity influencing growth resilience and a transmitter of temporal coherence directly influencing growth resilience. This dual role suggests that cognitive curiosity may be an important hub connecting cognitive resources and behavioural adaptations. When individuals possess clear goals, this goal-orientation translates into a desire to explore paths to achieve them; and when individuals have a balanced view of time, this cognitive trait inherently involves openness to new experiences, which together contribute to growth resilience by stimulating cognitive curiosity [19].

### 4.4 Theoretical contributions and practical implications

The theoretical contributions of this study are mainly reflected in three aspects: firstly, by introducing the concept of temporal coherence, it enriches the theoretical system of growth resilience antecedent variables, and expands the research perspective from the traditional personality traits to the field of temporal cognition; secondly [20], by constructing the chain mediation model, it reveals the intrinsic mechanism of the action from temporal cognition to mental resilience, and breaks the limitation of previous studies that mostly focus on the direct effect; and lastly. The central role of cognitive curiosity in the development of mental toughness was discovered, providing a new theoretical perspective for understanding individuals’ positive adaptation in adversity [21].

At the practical level, this study provides a clear direction for mental health education and resilience development for college students [22]. Intervention programs could be designed to target each component of the identified pathway sequentially. First, time perspective interventions could help students develop a more balanced and coherent temporal outlook. Second, goal-management workshops could assist students in formulating clear, meaningful personal and academic goals. Third, and crucially, educators should actively nurture cognitive curiosity. This can be achieved by incorporating inquiry-based and problem-based learning pedagogies, creating environments that reward exploration and tolerate uncertainty, and encouraging reflective practices that connect new knowledge to personal goals. Psychological counseling could also assess and support students’ motivational states, including their levels of curiosity [23].

### 4.5 Research limitations and future directions

This study has several limitations that need to be improved in future research.First, the cross-sectional design precludes definitive causal inferences. Future research should employ longitudinal designs to track the temporal dynamics of these constructs or experimental interventions to test causal effects [24]. Second, the self-report method was used for all data, which may have a common methodological bias, and although statistical tests showed that the problem was not serious, future studies could incorporate a variety of data sources, such as behavioural observations and peer evaluations. Third, the sample consisted solely of Chinese university students, limiting the cultural generalizability of the findings. Cross-cultural replications and extensions to other age or demographic groups are warranted [24].

Beyond addressing these methodological limitations, future research could: (a) investigate potential moderators (e.g., personality traits, social support) that may strengthen or weaken the identified mediation pathways; (b) examine whether specific sub-dimensions of temporal coherence (e.g., future orientation vs. past-positive) differentially predict resilience; and (c) develop and evaluate theory-driven interventions based on this sequential model to directly test its utility in enhancing student resilience [22].Methodologically, future research could incorporate multi-source data (e.g., behavioral observations, teacher or peer ratings) or experience sampling methods to reduce common method bias and capture the dynamic nature of these psychological processes over time.

## 5 Conclusions

This study yields three main conclusions. First, temporal coherence promotes college students’ growth resilience both directly and through multiple indirect pathways. second, goal clarity and cognitive curiosity act as chain mediators between temporal coherence and resilience, and this pathway is the main mechanism explaining the relationship between the two; Third, cognitive curiosity emerged as the strongest proximal predictor and appears to be a core driver in the development of growth resilience. Collectively, these findings illuminate a sequential cognitive-motivational pathway from coherent time perception to resilient growth. They offer a theoretical framework for understanding resilience and provide actionable insights for designing interventions aimed at fostering psychological adaptation and growth in college students.

## Supporting information

S1 Data Excel file containing the raw data used in this study. (XLSX)
